# Aortic Dissection Mimicking Myocardial Infarction: The Diagnostic Value of Point-of-Care Echocardiography

**DOI:** 10.7759/cureus.102217

**Published:** 2026-01-24

**Authors:** Grahith Pai Baidebettu, Ayman Helal, Mohammed El-din, Ibrahim Antoun

**Affiliations:** 1 Internal Medicine, Kettering General Hospital, Kettering, GBR; 2 Cardiology, Kettering General Hospital, Kettering, GBR

**Keywords:** aortic dissection, bedside echocardiography, diagnostic challenge, point-of-care-ultrasound, stemi mimickers

## Abstract

Aortic dissection (AD) is a life-threatening condition often misdiagnosed due to its varied presentation and symptom overlap with more common emergencies. This delay in diagnosis significantly increases morbidity and mortality. We report the case of a 53-year-old man with a history of hypertension who presented with central chest pain and was initially diagnosed with an inferior ST elevation myocardial infarction based on electrocardiographic findings. During cardiac catheterisation, difficulty in engaging the right coronary artery was noted, prompting a bedside transthoracic echocardiogram (TTE), which revealed a Stanford Type A AD extending from the aortic root to the abdominal aorta. The patient experienced ventricular fibrillation and cardiac arrest but was successfully resuscitated and immediately transferred for surgical repair. His postoperative course was complicated by multifocal cerebral infarcts requiring prolonged ventilatory support and tracheostomy.

This case highlights the crucial importance of maintaining a high clinical suspicion for AD, particularly when initial presentations are atypical or defy conventional diagnostic algorithms. We emphasise the pivotal role of early point-of-care echocardiography in rapidly confirming the diagnosis, thereby reducing delays to life-saving interventions. This significantly improves patient outcomes even in complex clinical scenarios. We also provide a practical guide for frontline clinicians to diagnose Type A AD using transthoracic echocardiography.

## Introduction

Cardiovascular disease is becoming a healthcare challenge, especially in the developing world [1-3]. Aortic dissection (AD) carries a high in-hospital mortality rate of 27.4%, with markedly worse outcomes in unoperated Stanford Type A dissections (58%) compared with unoperated Type B dissections (10.7%), as reported in a large registry study [4]. AD affects 4000 people every year in the UK [5]. Diagnosis of AD is often delayed as chest pain is traditionally associated with acute coronary syndrome and pulmonary embolism. Owing to its low incidence, aortic dissection is rarely encountered in routine clinical practice [6]. A delay in diagnosis of AD progressively increases the risk of complications, including aortic rupture, tamponade, and acute heart failure secondary to aortic valve regurgitation or coronary ischemia [7]. AD is diagnosed via imaging such as computed tomography angiography (CTA) or a transthoracic echocardiogram (TTE). Although transthoracic echocardiography is not a routine component of standard chest pain or acute coronary syndrome algorithms and is recommended mainly as an adjunct in cases of diagnostic uncertainty or suspected complications where reperfusion is not delayed, its use is increasingly being integrated into early diagnostic pathways in many centres. Acute AD is often classified into Stanford Type A and Type B, with the former needing emergency surgery. We describe a case of a middle-aged man presenting with chest pain, initially diagnosed as an inferior ST elevation myocardial infarction (STEMI). However, a bedside TTE suggested Stanford Type A AD on all views. The patient was then promptly transferred for surgical repair. We also present a guide for clinicians on diagnosing AD using TTE.

## Case presentation

**Figure 1:**
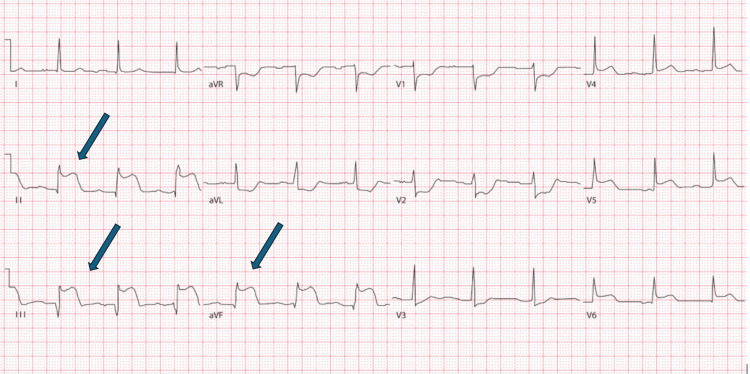
A 12-lead electrocardiogram, demonstrating ST elevation in leads II, III and aVF (blue arrows)

A 53-year-old man, a lifelong non-smoker with a history of hypertension (well managed with Ramipril 5 mg) with no significant cardiac family history, presented to the hospital with a complaint of central crushing chest pain for an hour that was non-radiating. The ambulance crew’s 12-lead electrocardiogram (ECG) demonstrated ST elevation in leads II, III, aVF, V5, and V6 (Figure 1). On arrival, he was hemodynamically stable with no inter-arm blood pressure variability. His troponin T level measured at presentation was later found to be 30.9 ng/L (reference range, <14 ng/L). He was diagnosed with an inferior ST elevation myocardial infarction, received standard initial acute coronary syndrome treatment with loading doses of dual antiplatelet agents, including aspirin 300 mg and ticagrelor 180 mg, and was transferred directly to the cardiac catheterisation laboratory (cath lab) for primary percutaneous coronary intervention.

**Figure 2:**
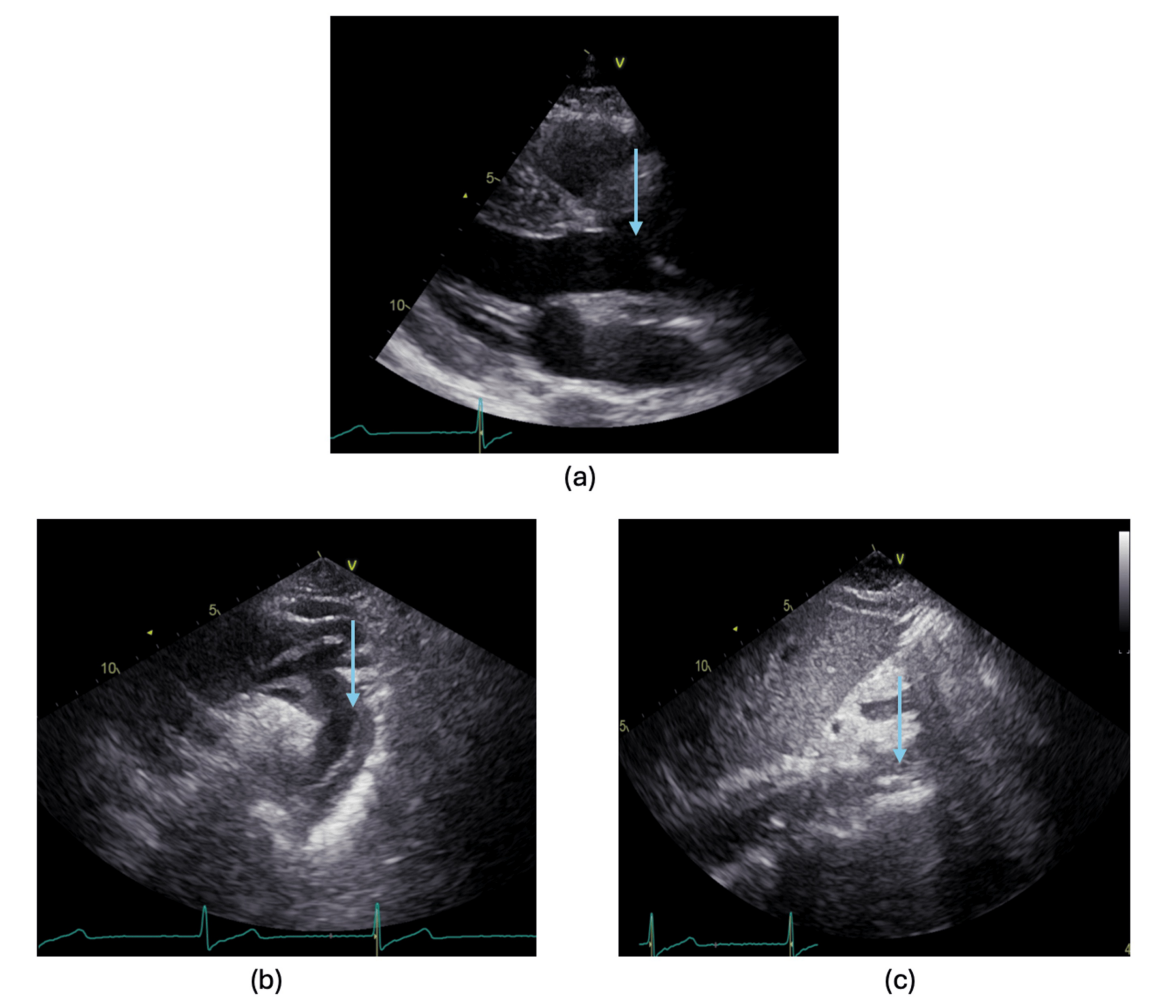
Echocardiograph images demonstrating aortic dissection (a) Parasternal long-axis echocardiographic view demonstrating a dissection flap at the aortic root level (blue arrow); (b) suprasternal echocardiographic view showing aortic dissection at the aortic arch level and descending aorta (blue arrow); (c) subcostal echocardiographic view demonstrating aortic dissection at the abdominal aortic level (blue arrow).

The decision was made to start with the right coronary artery (RCA) as it was the likely culprit. During the procedure, attempts to engage the RCA were complicated by pressure dampening of the arterial waveform, suggesting ostial compromise, and abnormal catheter movement within the aortic root raised suspicion of an underlying AD. At this point, 45 minutes had elapsed since hospital presentation, and a bedside transthoracic echocardiogram was performed, demonstrating a dilated aortic root with a dissection flap on all echocardiographic views, including the aortic root and ascending aorta on the long axis, aortic arch, and descending aorta on the suprasternal view extending to the abdominal aorta in the subcostal view (Figure 2). The patient’s heart went into ventricular fibrillation, followed by cardiac arrest. He was resuscitated using a direct current shock.

The patient was transferred to a tertiary care centre; CTA of the aorta was organised for surgical planning, which showed a large aortic dissection originating from the aortic root, extending to the left common iliac artery. He was then taken to the operating theatre, with surgical intervention commencing within four hours of presentation, where he had aortic root repair, ascending aortic replacement, and an RCA bypass graft. A 20-minute period of circulatory arrest was undertaken under moderate hypothermia (28 °C), with retrograde cerebral perfusion for cerebral protection.

**Figure 3:**
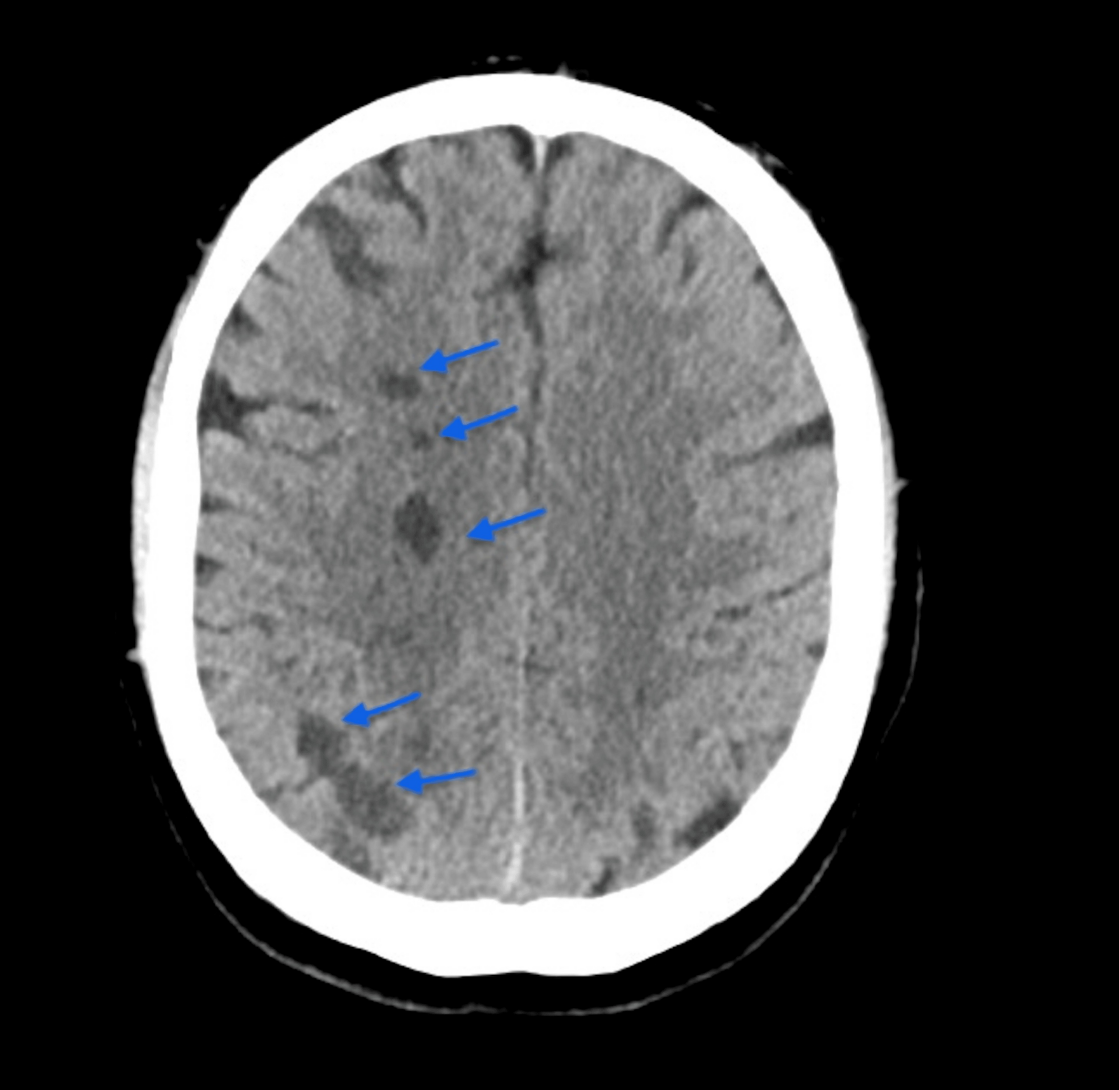
Head CT demonstrating multifocal infarcts within the right cerebral hemisphere (blue arrows)

The postoperative recovery phase was quite complicated. The patient developed a seizure; a head CT scan was ordered, which showed multifocal infarcts involving the right cerebral hemisphere, likely embolic in origin (Figure 3). After a discussion with the stroke team, he was managed conservatively with aspirin and intermittent pneumatic compression stockings. Low molecular weight heparin venous thromboembolism prophylaxis was deemed a contraindication and was withheld to prevent the haemorrhagic transformation of stroke. He had a difficult extubation and went on to require a temporary tracheostomy. Postoperative echo showed mildly impaired right ventricular function with a left ventricular ejection fraction of >50%. He was transferred to a stroke ward where he received neurorehabilitation. The patient was subsequently discharged and followed up for two years, during which he showed sustained clinical improvement.

## Discussion

Presenting symptoms of AD can range from asymptomatic presentation to the classical presentation of sudden tearing chest pain radiating to the back. The most common risk factors for AD are atherosclerosis and arterial hypertension in the elderly, followed by connective tissue diseases (Marfan syndrome, Ehlers-Danlos syndrome) in the young, bicuspid aortic valves, and recreational drug usage (cocaine) [5,8]. A thorough family history is essential, as a first-degree relative with AD increases an individual’s risk by 2.77-fold [9]. Inherited conditions, such as familial thoracic aortic aneurysm and dissection (FTAAD), often follow an autosomal dominant pattern, underscoring the importance of genetic risk assessment. The Stanford system classifies ADs as Type A (involving the ascending aorta) or Type B (occurring distal to the subclavian artery), while the DeBakey system categorizes them as Type I (beginning in the ascending aorta and extending to the descending), Type II (confined to the ascending aorta), or Type III (starting in the descending aorta distal to the left subclavian artery) [10]. Stanford Type A or DeBakey Type I and II are cardiovascular emergencies and warrant emergency surgical repair to prevent fatal complications [11]. The mortality rate of untreated AD can be as high as 1% per hour due to the delay in management [12].

There have been many reported cases of AD being misdiagnosed as myocardial infarction [13]. When AD leads to myocardial infarction, it is typically because the dissection flap cuts off blood supply to the coronary arteries, or because a clot that has formed in the false lumen presses on and narrows the true coronary lumen [14]. Misdiagnosis carries significant risks, particularly when antiplatelet or thrombolytic agents are administered before definitive surgical repair of AD. These interventions may exacerbate bleeding complications like cardiac tamponade (which is the most common cause of death from proximal AD [15]), reinforcing the critical importance of accurate early diagnosis. One case report highlights the use of percutaneous coronary intervention for initial stabilisation in patients experiencing myocardial infarction due to AD; however, this approach warrants further exploration [16]. Although CTA is considered the gold standard for accurate assessment of aortic anatomy with sensitivity ranging from 94% to 100%, it is not without its limitations. There have been cases of missed Type A AD even on CTA [17].

TTE allows for precise and quick evaluation of the aortic root and proximal ascending aorta and, in most cases, also enables assessment of the aortic arch, proximal descending aorta, and abdominal aorta. Table 1 shows the segments of the aorta and the optimal viewing windows for each segment [18].

**Table 1:** Correlation between echocardiographic windows and the corresponding segments of the aorta Created by the authors.

Echocardiographic View	Segments of the Aorta
Left parasternal long-axis view	Aortic root proximal ascending aorta
Apical three chamber	Proximal ascending aorta
Apical five chamber	
Modified subcostal view (in children)	
Upper intercostal spaces	Mid- and distal ascending aorta
Right parasternal view	Distal ascending aorta
Suprasternal view	Aortic arch proximal descending aorta
Parasternal long-axis view	Descending aorta

**Figure 4:**
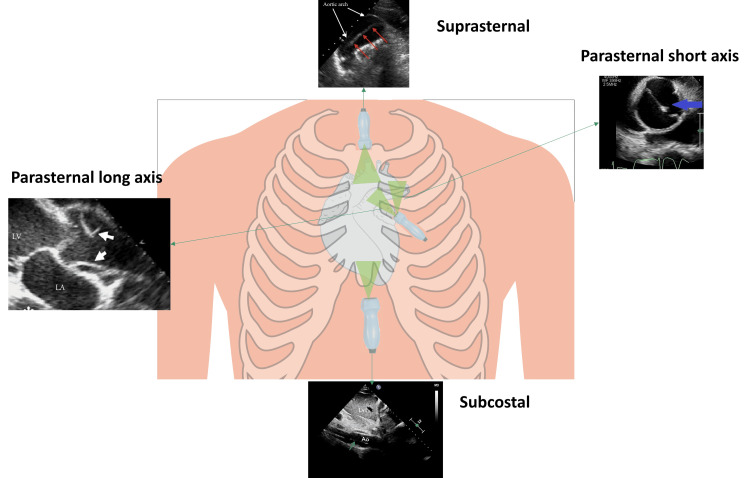
Echocardiographic windows and their corresponding imaging perspectives. Parasternal long-axis, parasternal short-axis, suprasternal, and subcostal views are shown, highlighting their role in visualising cardiac and aortic structures. Arrows denote the aortic dissection, with white arrows in the parasternal long-axis view, blue arrows in the parasternal short-axis view, red arrows in the suprasternal view, and green arrows in the subcostal view. A suprasternal view can be obtained by positioning the transducer in the suprasternal notch, angling it towards the patient's left shoulder. To get the parasternal long-axis view (PLAX) view, place the transducer on the left chest, next to the sternum in the third or fourth intercostal space with the probe facing the right shoulder and adjust until the heart is visible. If we rotate the probe by 90 degrees, with the marker pointing to the patient's left shoulder, we can obtain a parasternal short-axis view (PSAX). Subcostal views can be obtained by positioning the transducer below the xiphoid process, with the marker facing the patient's left side, angling the beam slightly anteriorly. Image created by the authors.

The sensitivity of TTE in the diagnosis of Type A AD is 78% to 90%, but only 31% to 55% in Type B dissection. Type A AD specificity ranges from 87% to 96%, and Type B dissection ranges from 60% to 83% [11]. Contrast can also be administered to visualise aortic anatomy better, increasing the sensitivity of TTE to 85% [19]. Performing bedside TTE saves crucial time in diagnosing Type A AD, potentially preventing hemodynamically unstable patients from being moved to a CT room. Another advantage is that TTE without contrast can be used in patients with low renal function. A guide to diagnosing Type A dissection on TTE for clinicians is shown in Figure 4.

However, it is essential to note that TTE has a low negative predictive value, which means that we cannot rule out dissection solely based on TTE; further imaging with CTA will be required if the exam is negative.

A key novelty of this case is the systematic echocardiographic approach used to identify AD. We provide a structured echocardiographic guide tailored for emergency and interventional settings, highlighting key sonographic features such as the intimal flap in multiple imaging planes, aortic root dilatation, and extension of the dissection into the descending aorta on suprasternal and subcostal views (Figure 4). While previous studies, such as the EASY [20], FATE [21], and FEEL [22] protocols, have demonstrated the utility of echocardiography, they tend to be exhaustive and require resource-intensive additional training. Moreover, they are not specifically tailored to the diagnosis of AD. Our guide consolidates the essential transthoracic echocardiographic views into a concise, one-page bedside reference. It is designed for clinicians already trained in transthoracic echocardiography, enabling them to quickly and effectively rule in AD without the need for further specialised training. Additionally, we discuss the evolving role of contrast-enhanced TTE, 3D/4D echocardiography, and AI-driven image interpretation, which are expected further to refine the echocardiographic evaluation of acute aortic syndromes.

Another distinctive aspect of this case is the complex postoperative course, including multifocal cerebral infarcts and the requirement for prolonged ventilatory support and tracheostomy. While surgical repair is the definitive treatment for Type A AD, the impact of perioperative complications on long-term outcomes remains underreported in similar cases. This highlights the need for multidisciplinary coordination, particularly between cardiology, cardiothoracic surgery, neurology, and critical care teams, to optimise patient outcomes.

## Conclusions

A high index of clinical suspicion is necessary for prompt diagnosis of AD. A wide spectrum of presenting symptoms of AD and its overlap with other common cardiac conditions make it a diagnostic conundrum. TTE can rule in dissection without waiting for CTA to save time and set things in motion for potential surgery. Advancements in resolution in point-of-care ultrasound and echocardiography, as well as the introduction of 3D and 4D TTE, are expected to alter many of the established diagnostic pathways in medical emergencies.
